# Integrated Gradient-Based Continuous Wavelet Transform for Bearing Fault Diagnosis

**DOI:** 10.3390/s22228760

**Published:** 2022-11-12

**Authors:** Junfei Du, Xinyu Li, Yiping Gao, Liang Gao

**Affiliations:** State Key Laboratory of Digital Manufacturing Equipment and Technology, School of Mechanical Science and Engineering, Huazhong University of Science and Technology, Wuhan 430074, China

**Keywords:** fault diagnosis, integrated gradients, continuous wavelet transform, convolutional neural networks

## Abstract

Bearing fault diagnosis is important to ensure safe operation and reduce loss for most rotating machinery. In recent years, deep learning (DL) has been widely used for bearing fault diagnosis and has achieved excellent results. Continuous wavelet transform (CWT), which can convert original sensor data to time–frequency images, is often used to preprocess vibration data for the DL model. However, in time–frequency images, some frequency components may be important, and some may be unimportant for DL models for fault diagnosis. So, how to choose a frequency range of important frequency components is needed for CWT. In this paper, an Integrated Gradient-based continuous wavelet transform (IG-CWT) method is proposed to address this issue. Through IG-CWT, the important frequency components and the component frequency range can be detected and used for data preprocessing. To verify our method, experiments are conducted on four famous bearing datasets using 3 DL models, separately, and compared with CWT, and the results are compared with the original CWT. The comparisons show that the proposed IG-CWT can achieve higher fault diagnosis accuracy.

## 1. Introduction

Bearings are widely used in machinery systems. The reliability of bearings is very important for ensuring safe operation and reducing losses for machinery systems [1,2]. However, most of bearings work under severe conditions, including high temperature, high rotating speed, high torque, etc. [3]. As a result, it is necessary to detect faults efficiently and accurately. Thus, it is of great importance to develop accurate fault diagnosis methods for fault diagnosis. Traditionally, fault diagnosis usually consists of three main phases, which are signal acquisition, feature extraction, and classification. There are some limitations of traditional fault methods (e.g., model-based method) that should be considered. Traditional methods need selecting features manually, which is usually time consuming, especially when dealing with large amounts of data. Additionally, if the selected features are inadequate for the task, the performance of the fault diagnosis will be greatly degenerated [4].

Alternatively, deep learning (DL)-based methods provide an end-to-end solution to overcome these limitations. DL models can learn hierarchy features and correlations among data automatically [5,6], which could avoid handcrafted feature selection. Nowadays, DL has been used widely in fault diagnosis [7,8] due to its powerful feature learning ability. Despite different kinds of neural networks being used in fault diagnosis, such as deep belief network (DBN) [9], autoencoder (AE) [10], and convolutional neural network (CNN) [11,12,13,14], in these DL methods, CNN models are the most widely used. Yang et al. [13] proposed an enhanced deep-CNN-based fuzzy fusion rotating machinery fault diagnosis method using three popular data preprocessing methods. Cheng et al. [15] proposed a fault diagnosis method combining a local binary CNN model and continuous wavelet transform for rotating machinery. Fang et al. [16] proposed a lightweight efficient extraction method based on CNN for bearing fault diagnosis. Ji et al. [17] proposed a two-stage method using order-tracking and one-dimensional CNN to deal with the problem of fault diagnosis under variable speed conditions. Bertocco et al. [18] presented a method based on CNN for classifying roller bearing failures, so to perform a predictive maintenance paradigm with a condition monitoring system. Gao et al. [19] developed a hierarchical training CNN for imbalanced fault diagnosis in complex equipment.

Although CNN models have the powerful ability to extract features automatically, in order to use vibration signal better, signal preprocessing methods are often used to transform a measured signal into time, frequency, and time–frequency domain [20]. Although time and frequency domain analysis methods are often used for fault diagnosis, neither of these methods can address signal variations in the association between time and frequency domains. In practical cases, most vibration signals from rotating machinery are non-stationary. Therefore, the vibration signal usually is transformed into time–frequency domain for fault diagnosis, for example, CWT [2,14,15], discrete wavelet transform (DWT) [21], short-time Fourier transform [22], and other methods [23,24,25]. Additionally, some studies show that time–frequency analysis methods have better performance. For example, Pandhare et al. [26] compared time, frequency, and time–frequency feature using a CNN model, and the results show that time–frequency features had better performance.

Continuous wavelet transform (CWT) is a classic time–frequency analysis method, which can be calculate by the inner product of signal and wavelet bases [27]. CWT has been widely used as a data preprocessing method for CNN-based fault diagnosis. For CWT, the sampling frequency and the decomposition scale are determined in advance [28]. In previous studies, expert’s knowledge was needed to determine the decomposition scale (corresponding to frequency range) of CWT. Nevertheless, the fault diagnosis strategies of the CNN model may not be consistent with expert’s knowledge, so may all frequency components not be important for fault diagnosis using a CNN model. Our interest in this work is exploring how to select the frequency range of important components without experts to augment CWT for fault diagnosis. Since the frequency resolution is limited, fewer unimportant frequency components mean more important frequency components, and the CNN-based model may benefit from this. So, CNN-based fault diagnosis methods would benefit from the frequency range of important frequency components for CWT. The CNN models are thought to be black boxes, and it is hard to know which frequency components are more important [29], however, the feature-attribution-based explainable method, one of the most popular explainable DL techniques for explaining image classification models, could show which features in an image are important [30]. So explainable DL may be a solution for the selection of frequency range for data preprocessing methods of CWT.

Recently, lots of local explanation methods have been proposed to visualize learned features and explore how they contribute to class predictions, such as Integrated Gradients (IG) [31], Shaply values [32], Class Activation Map (CAM) [33], Gradient-weighted Class Activation Map (Grad-CAM) [34], etc. [35,36,37]. Some explainable DL methods have been used for fault diagnosis. Kim and Kim [38] analyzed the feature representation of the trained CNN for bearing fault diagnosis using the Grad-CAM method. Grezmak et al. [39] proposed an explainable deep CNN model for fault diagnosis of gearboxes. However, in this study, we try to use explainable DL techniques to extract the importance weight of input features for fault diagnosis. Attribution-based methods focus on explaining how a DNN mode makes decisions [32] by pointing out the importance of input features, which satisfies the requirement of finding important frequency components. Integrated Gradients [31] is an axiomatic model interpretability algorithm that assigns an importance score to each input feature by approximating the integral of gradients of the model’s output with respect to the inputs along the path from given baselines to inputs. SmoothGrad (SG) [40] seeks to alleviate noise and visual diffusion for saliency maps by averaging over explanations of noisy copies of an input. In this study, IG and SG are used to extract the importance weight of frequency components for selecting the frequency range.

Based on the explainable DL method IG, we design an Integrated Gradient-based continuous wavelet transform (IG-CWT) method for data preprocessing for fault diagnosis. The importance weight of frequency components is obtained by using IG and SG, then a frequency range of important frequency components is generated based on the important weight. The frequency range is used for CWT to convert the data into time–frequency images as the preprocessed data.

The rest of the paper is organized as follow. Section 2 presents our proposed method IG-CWT, including CWT and the method for generating the frequency range. Section 3 presents the testing results of IG-CWT. Conclusions and future work are presented in Section 4.

## 2. Framework of the Proposed IG-CWT

**Figure 1:**
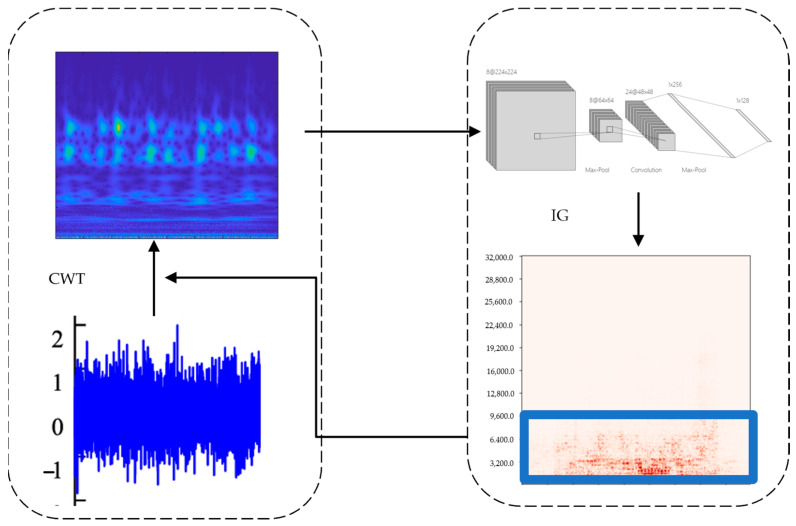
Framework of IG-CWT.

In this section, the framework of the proposed method IG-CWT is demonstrated. The method can select the important frequency range and generate time–frequency images for fault diagnosis, which consists of the following steps, as shown in Figure 1; step 1 original vibration signals are converted into time–frequency images by CWT, with frequencies range from 0 to half of sampling frequency; step 2, the CNN model is trained using these images; step 3, the feature attribute method IG is used to obtain the importance score of the frequency components, and the frequency range is obtained based on the feature importance scores; and step 4, CWT is used again to transform the original signals into time–frequency images; with the frequency range obtained in step 3, we obtain the final preprocessed data. The details are demonstrated in the subsections bellow.

### 2.1. Continuous Wavelet Transform

CWT is widely used as a data preprocessing method to extract signal features in the time domain and corresponding spectral content in the frequency domain. CWT is used to convert original signals into time–frequency distributions, generating representations of the original signal in the time and frequency domains simultaneously [41] in the form of time–frequency images.

CWT conducts an inner product operation of the signal and a set of wavelets, which is called a wavelet family [27]. A wavelet family is generated by scaling and translating; the mother wavelet is defined as:

(1) $$\psi_{s,\tau }=\frac{1}{\sqrt{s}}\psi \left(\frac{t-\tau }{s}\right)$$

where *s* is the scale parameter, τ is the translation parameter, and *s* is related to frequency inversely [42].

To obtain the CWT of a given signal *x*(*t*), a convolution operation of a complex conjugate can be conducted, which is mathematically defined as follows:

(2) $$W\left(s,\tau \right)=\langle x\left(t\right),\psi_{s,\tau }\rangle =\frac{1}{\sqrt{s}}\int x\left(t\right)\psi^{*}\left(\frac{t-\tau }{s}\right)dt$$

where ψ* represents the complex conjugate. This equation demonstrates that the CWT is similar to the Fourier transform, where a signal can be decomposed into the frequencies that it is composed of [14]. Through this equation, the signal *x*(*t*) is decomposed into a series of wavelet coefficients, where the wavelet family is the basis function. Based on above equations, there are two kinds of parameters in family wavelets: s and τ.

After the convolution operation, the signal *x*(*t*) is transformed by the family wavelets and projected to the two-dimensional (2-D) time and scale (or frequency) dimensions [41]. In this way, one-dimensional time series are converted to time–frequency images. The frequency range is related by chosen scales.

### 2.2. IG-Based Frequency Range Selection

In our formal setup, an input is a vector $x\in R^{d}$. A model describes a function *S*: $R^{d}\to R^{c}$, where *C* is the number of classes which donate the fault classes. An explainable method provides an explanation map *E*: $R^{d}\to R^{d}$ that maps inputs to objects of the same shape. Where the explanation map *E* donates the feature importance of input time–frequency images for the classification result. Feature attribution methods are among the most popular techniques for explaining image classification models because they can show which pixels in an image are important. In other words, the feature attribution explanations have the same resolution with the inputs without up-sampling. In this study, IG is used to obtain attribution explanations and SG is used to alleviate noise.

Integrated Gradients (IG) also addresses gradient saturation by summing over scaled versions of the input [31]. IG considers the straight-line path from the baseline $\bar{x}$ to the input *x* and computes the gradients at all points along the path. Integrated Gradients are obtained by cumulating these gradients. IG for an input *x* is defined as:

(3) $$E_{IG}\left(X\right)=\left(x-\bar{x}\right)\int_{0}^{1}\frac{\partial S\left(\bar{x}+\alpha \left(x-\bar{x}\right)\right)}{\partial x}d\alpha$$

where $E_{IG}$ is the attribution explanation of IG, $\bar{x}$ is a baseline input that represents the absence of a feature in the original input *x*, and ∂s/∂x is the gradient of *S*.

SmoothGrad (SG) [40] seeks to alleviate noise and visual diffusion for explanation by averaging over explanations of noisy copies of an input. For an input *x,* random samples in a neighborhood of input are taken and average the resulting attribution explanation maps. For a given explanation map *E*, SG is defined as:

(4) $$E_{SG}\left(x\right)=\frac{1}{N}\sum_{i=1}^{N}E\left(x+g_{i}\right)$$

where noise vectors $g_{i}∼N\left(0,\sigma^{2}\right)$ are drawn from a normal distribution with standard deviation σ, *N* is the number of samples, and $E_{SG}$ is attribution of SG.

Since these methods are local explainable methods, one explanation is for one input, and the one explanation is one feature importance map for the corresponding input. The samples in the test set are used to generate explanations. We obtain the same number of explanations as samples in the test set. Since one explanation map is for one input, in order to choose the important frequency components, the frequency importance score is defined as the average of all attributions of all time point samples:

(5) $$W_{f}=\frac{1}{N\times T}\sum_{N}\sum_{t=0}^{T}E_{f,t}$$

where *N* is the amount of input samples in the test set, *T* is the amount of time points, $E_{f,t}$ is the feature importance of feature in *f* row and *t* column in the time–frequency image, and *f* is the frequency. We introduce a frequency importance threshold *λ* to choose important frequencies. If $W_{f}\geq \lambda \times average\left(W_{f}\right)$, we consider that the *f* is an important frequency, defined as $f_{i}$. These important frequencies form the important frequency set {$f_{i}$}, and the important frequency range is defined as minimum frequency to maximum frequency in {$f_{i}$}:

(6) $$F_{range}=min\left\{f_{i}\right\}\;\sim \;max\left\{f_{i}\right\}$$

where $F_{range}$ is the frequency range from minimum frequency to maximum frequency in {$f_{i}$}, and $min\left\{f_{i}\right\}\;$and $max\left\{f_{i}\right\}$ are minimum and maximum frequency of $\left\{f_{i}\right\}$, respectively.

Finally, CWT is conduct using the frequency range to generate the final preprocessed time–frequency images. In the next section, we test the performance of different *λ* values and give the recommended value of *λ*.

## 3. Experimental Results and Discussion

To verify the performances of our proposed data preprocessing method IG-CWT for fault diagnosis, we conduct experiments on four open bearing dataset, which are the bearing fault datasets of Paderborn University (PU), Society for Machinery Failure Prevention Technology (MFPT) dataset, Jiangnan University (JNU) bearing dataset and Case Western Reserve University (CWRU) dataset. Additionally, three CNN models, AlexNet, ResNet18, and VGG16, are used for the experiments because the three models, or the models based on them, are used the most frequently in the fault diagnosis literature. For each dataset, comparative experiments between IG-CWT and CWT are conducted to verify the effectiveness of IG-CWT using the three models above. Additionally, in order to obtain the best value of hyperparameter *λ*, comparative experiments are carried out with different values of hyperparameter *λ*. Classification accuracy is calculated as the metrics [43] to evaluate the performance of IG-CWT, which is defined as the proportion of samples which are right classified to all samples:

(7) $$Accuracy=\frac{n_{right}}{N_{total}}$$

where $n_{right}$ is the number of samples classified rightly in the testing set, and $N_{total}$ is the total number of samples in the testing set. Ten runs are performed, and the average accuracy is considered as the result.

### 3.1. PU Dataset

**Figure 2:**
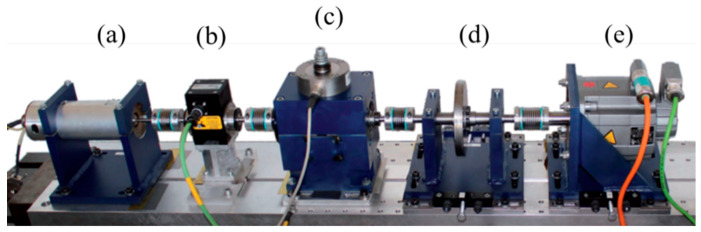
Modular test rig of PU dataset.

The Paderborn University (PU) dataset is provided by the Paderborn University Bearing Data Center [44,45]. The test ring of the PU dataset consists of several modules: an electric motor (a), a torque-measurement shaft (b), a rolling bearing test module (c), a flywheel (d), and a load motor (e); see Figure 2. There are three kinds of bearings in the PU dataset: (1) six undamaged bearings; (2) twelve artificially damaged bearings; and (3) fourteen bearings with real damages caused by accelerated lifetime tests. To show the performance of the proposed preprocessing method, we would like to use the data collected from real damaged bearings (including KA04, KA15, KA16, KA22, KA30, KB23, KB24, KB27, KI14, KI16, KI17, KI18, and KI22). Since KI04 was the same as KI14, we kept KI04 [44]. Here, the details of the used fault dataset are described as follows: the bearing rotating speed is 1500 rpm; the load is 0.7 Nm; the radial force is 1000 N; and the sampling rate is 64 kHz. Hence, there is a 13-class classification task.

For data preparation, raw data is split into samples with 1024 data points. CWT is used to convert vibration data into time–frequency images, and scalars of CWT are set depended on the frequency components uniformly distributed over 0–32 kHz. Each sample is converted into a time–frequency image with a size of 224 × 1024, where 224 is the dimension of frequency and 1024 is the dimension of time. Then, the time–frequency images are resized to 224 × 224 for CNN models. There are 13 class samples, and 500 samples are used for each class. The samples are randomly divided into training, validation, and testing sets with the ratios of 0.6, 0.2, and 0.2. For CNN models, AlexNet, ResNet18, and VGG16 are used separately. The Adam is used with a learning rate 0.001, and the batch size is 64. After model training with CWT, IG-CWT is used to select frequency range and convert raw data into time–frequency images with the selected frequency range.

**Figure 3:**
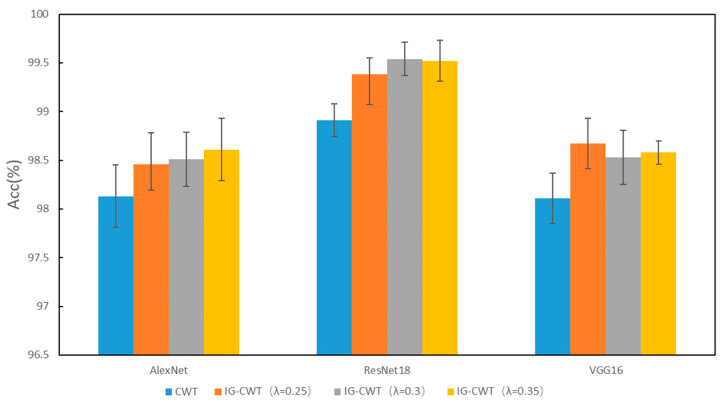
PU accuracy of IG-CWT and CWT.

To validate the effectiveness of our IG-CWT method, comparative experiments are carried out to compare classification accuracies of IG-CWT (with λ = 0.25, 0.3, and 0.35, respectively) and CWT. The comparison results are shown in Figure 3. The CWT in Figure 3 denotes using CWT with frequency range of 0–*f_s_*/2 (Nyquist frequency), where *f_s_* is sampling frequency. Although different models have different accuracies, IG-CWT (with *λ* = 0.25, 0.3, and 0.35, respectively) has better accuracy performance compared with CWT. Although there are only three models tested, many other CNN models for fault diagnosis are based on the three models; we believe that our IG-CWT method can be used for these CNN models.

**Figure 4:**
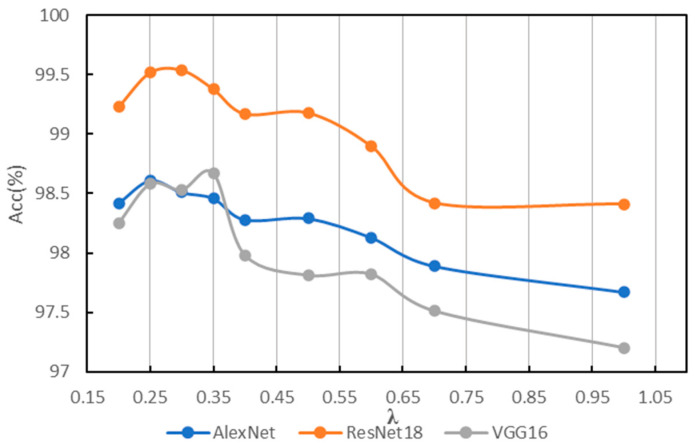
Results of different *λ* on PU.

In order to find a suitable hyperparameter *λ* of IG-CWT for data preprocessing, different values of hyperparameter *λ* are tested using the accuracy of fault diagnosis with the three models mentioned above. The *λ* is set as 0.1, 0.2, …, 1.0, and we found that close frequency ranges are obtained with 0.8, 0.9, and 1.0, so only 1.0 is kept. Additionally, when *λ* = 0.1, the frequency range is 0–32 kHz. So, 0.1 is discarded. The results based on ResNet18 are shown in Table 1; the results show that different frequency ranges are obtained with different values of *λ*, which have influence on the accuracy of fault diagnosis. When *λ* = 0.3, ResNet18 can achieve fault diagnosis accuracy of 99.54, which is the best accuracy of different values of λ in our experiments. So, a *λ* = 0.35 and 0.25 is added for experiments. We conduct the same experiments with AlexNet and VGG16; the results of the three models are in Table 1. For reading convenience, the results are visualized in Figure 4. The results show that different models have different obvious performance in accuracy with different frequency ranges. Results show that AlexNet, ResNet18, and VGG16 achieve the best classification performance with *λ* = 0.25, 0.3, and 0.35, respectively, the best accuracies are in bold in Table 1. Figure 4 indicates that when *λ* = 0.35~0.25, models have the best performance in general. Additionally, when *λ* < 0.25, the reason for lower accuracy is that some unimportant frequency features are incorporated in the input time–frequency images. When *λ* > 0.35, the reason for lower accuracy is that some important frequency features are not incorporated in input images. Additionally, although there are three values of *λ* for the best accuracies, they are in a small scope of 0.25~0.35, and the frequency ranges for the best accuracy are similar, which are 0.2–16.8, 0.4–17.0, and 0.2–16.6. So, a suitable frequency range is needed for fault diagnosis, even though models may be different.

### 3.2. MFPT Datasets

The Machinery Failure Prevention Technology (MFPT) dataset is provided by the Society for Machinery Failure Prevention Technology. The MFPT bearing dataset (artificial fault bearing dataset) contains three main health conditions: normal state, inner race fault state, and outer race fault state [46]. The normal-state data were gathered under a same load, the outer race fault state data were gathered under seven different loads, respectively, and the inner race fault state data were also gathered under seven different loads, respectively. Under different load, the same fault type would contain different loads, respectively. Under different load, the same fault type would contain different information. Hence, there are seven labels in the inner race fault state and outer race fault state. Therefore, there is a total of 15 labels in this case. It can be seen as a 15-class classification task. As for data files, the normal-state data were gathered at 97,656 Hz; seven kinds of outer ring fault data and seven kinds of inner ring fault data were collected as 48,828 Hz. Before data preprocessing, normal data are down-sampled to 48,828 Hz.

**Figure 5:**
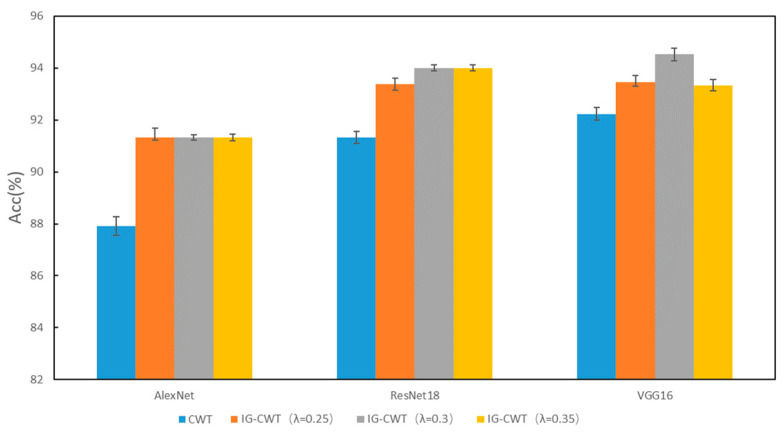
MFPT accuracy of IG-CWT and CWT.

For experiments on MFPT, vibration data were split into samples with 1024 data points, as the PU dataset. There are 143 samples for each class, and the samples are randomly divided into training, validation, and testing sets with the ratios of 0.6, 0.2, and 0.2. Similar experiments as with the PU dataset are conducted. Comparative experiments are carried out on the MFPT dataset to compare the classification accuracy of IG-CWT (with *λ* = 0.25, 0.3, and 0.35, respectively) and CWT. The results are shown in Figure 5. Our proposed method can achieve a higher fault diagnosis accuracy obviously with *λ* = 0.25, 0.3, and 0.35, respectively. This indicates our method is effective for data preprocessing for fault diagnosis.

**Figure 6:**
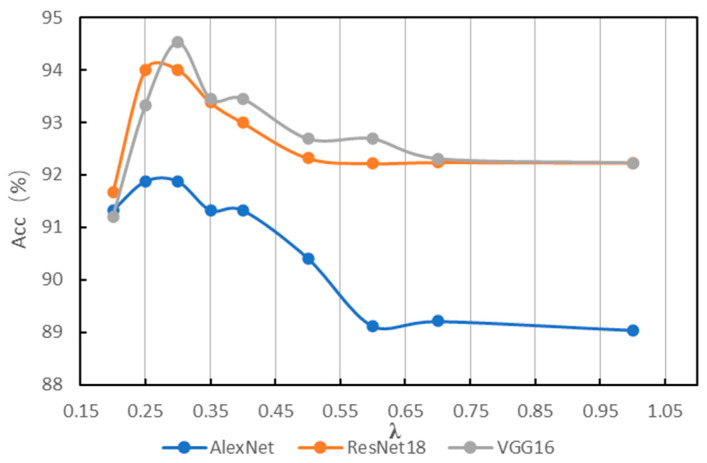
Results of different *λ* on MFPT.

The experiments with different values of *λ* are conducted on MFPT using similar settings as Section 3.1; the results are shown in Table 2 and Figure 6. As is shown in Figure 6, when *λ* = 0.25~0.35, models achieve better accuracies. The best accuracies are in bold in Table 2. Additionally, we find that in some cases different values of *λ* have the same frequency range, this is because the importance score of one frequency is much larger than the adjacent one. Additionally, the frequency ranges are similar for different models to achieve the best accuracies. This phenomenon indicates that accuracy of fault diagnosis benefits from a suitable frequency range.

### 3.3. JNU Bearing Dataset

The Jiangnan University (JNU) bearing dataset is provided by Jiangnan University [45]. The JNU dataset contains one health state and three fault modes, including inner ring fault, out ring fault, and rolling element fault. An accelerometer (PCB MA352A60) with a bandwidth from 5 Hz to 60 kHz and a 10 mV/g output is used to measure the vertical vibration signals in the health, inner ring fault, outer ring fault, and rolling element fault states, respectively. Additionally, the rotation speeds are 400, 600, and 800 rpm while the signals are measured. The sampling frequency of the signal measurement is 50 kHz, and the sampling time is 20 s. Therefore, the total number of classes was equal to twelve according to different working conditions.

**Figure 7:**
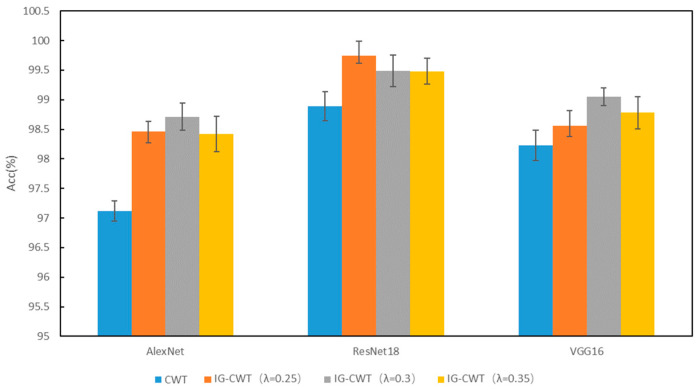
JNU accuracy of IG-CWT and CWT.

For experiments of the JNU bearing dataset, vibration data were split into samples with 1024 data points, as with the above experiments. There are 976 samples for each class, and the samples are randomly divided into training, validation, and testing sets with the ratios of 0.6, 0.2, and 0.2. Comparative experiments are carried out on the JNU dataset to compare the classification accuracy of IG-CWT (with *λ* = 0.25, 0.3, 0.35 respectively) and CWT. Results are shown in Figure 7. Our proposed method (with *λ* = 0.25, 0.3, and 0.35, respectively) can achieve a higher fault diagnosis accuracy obviously with different models. This indicates our method is effective for data preprocessing for fault diagnosis.

**Figure 8:**
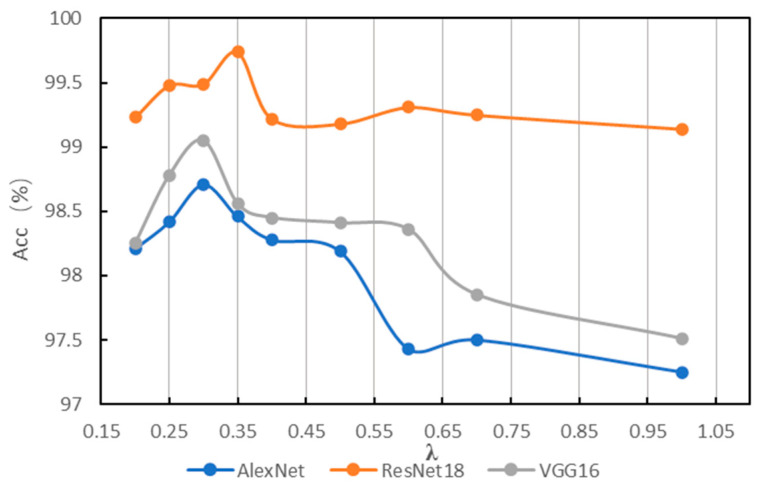
Results of different *λ* on JNU.

The experiments with different values of *λ* are conducted on the JNU dataset using similar settings as Section 3.1; the results are shown in Table 3 and Figure 8. As the results show, when *λ* = 0.3~0.35, models achieve the best classification accuracy, respectively the best accuracies are in bold in Table 3. This phenomenon is similar with the above two cases. Additionally, the frequency ranges are similar to the best accuracy with different models. This phenomenon indicates that the accuracy of fault diagnosis benefits from a suitable frequency range.

### 3.4. CWRU Bearing Dataset

The Case Western Reserve University (CWRU) bearing dataset is provided by the Case Western Reserve University Bearing Data Center [47]. In this dataset, there are three fault types and three fault diameters for each fault type. The three fault types are roller fault, outer race fault, and inner race fault. The fault diameters are 0.18, 0.36, and 0.54 mm. The drive-end vibration signals are collected under four different operational conditions with respect to different bearing loads (load 0–3 hp) with a sampling frequency of 12 kHz. Each operational condition has nine fault categories with one health state. So, there are totally ten health conditions.

**Figure 9:**
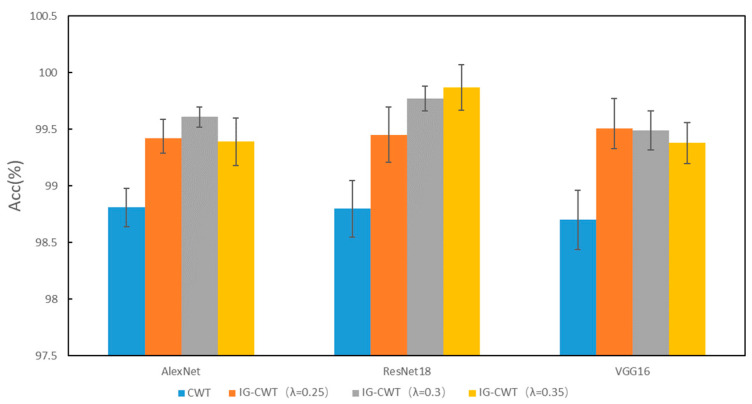
CWRU accuracy of IG-CWT and CWT.

For experiments on the CWRU bearing dataset, vibration data were split into samples with 1024 data points, as with the above experiments. There are 8400 samples totally and 840 samples with the four operational conditions mentioned above for each class, and the samples are randomly divided into training, validation, and testing sets with the ratios of 0.6, 0.2, and 0.2. To validate the effectiveness of our IG-CWT method, comparative experiments are carried out on the CWRU dataset to compare the classification accuracy of IG-CWT (with *λ* = 0.25, 0.3, and 0.35, respectively) and CWT. Results are shown in Figure 9. Our proposed method can achieve a higher fault diagnosis accuracy with *λ* = 0.25, 0.3, and 0.35 obviously with different models. This indicates our method is effective for data preprocessing for fault diagnosis.

**Figure 10:**
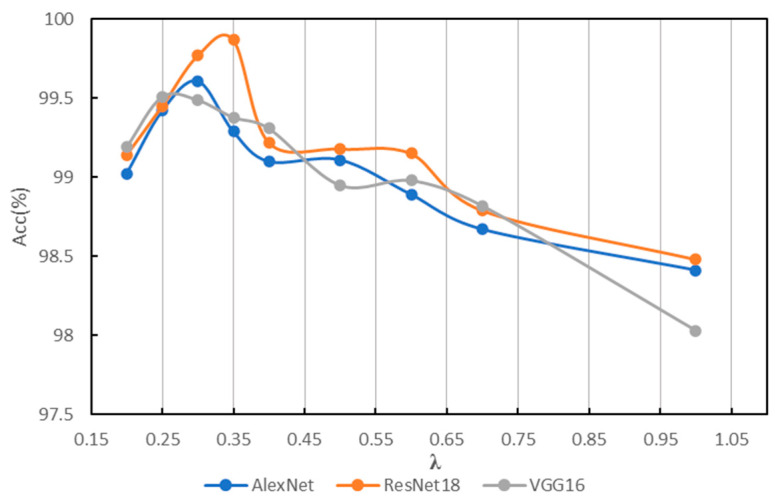
Results of different λ on CWRU.

In order to find a find suitable hyperparameter *λ* of IG-CWT for data preprocessing, different values of hyperparameter *λ* are tested using the accuracy of fault diagnosis with three models. The experiment’s setting is similar to Section 3.1, the results are shown in Table 4 and Figure 10. As the results show, when *λ* = 0.25~0.35, models achieve the best accuracy, respectively, the best accuracies are in bold in Table 4. This phenomenon is similar to the other three cases. Additionally, the frequency ranges are similar to the best accuracy with different models. This phenomenon indicates that the accuracy of fault diagnosis benefits from a suitable frequency range.

### 3.5. Discussion

In the case studies, the effectiveness of our proposed IG-CWT method is verified on four bearing datasets; the prediction accuracies of our proposed method are much higher than CWT, respectively. Additionally, in this section, experiments are conducted using different values of hyperparameter *λ* to find a suitable scope of *λ*. Although different models achieve their best accuracy at different λ, the values of λ with the best performance are in a small scope, and results of experiments show that *λ* = 0.25~0.35 is suitable for bearing fault diagnosis. Meanwhile, the frequency ranges are similar when achieving the best accuracies; this indicate that a suitable frequency range for data preprocessing is needed for fault diagnosis. When IG-CWT is used for fault diagnosis, the CNN model will be trained once more compared with CWT, so it has higher time and space complexity. Additionally, the extra time and space complexity of IG-CWT is roughly the same as the CNN model used for fault diagnosis.

## 4. Conclusions and Future Research

In conclusion, an IG-based data preprocessing method IG-CWT for bearing fault diagnosis is proposed in this paper, and we conducted experiments to validate our proposed method. In the IG-CWT method, the parameter λ is introduced in our method for finding the suitable frequency range. Additionally, *λ* is suggested to be set to 0.25~0.35 for fault diagnosis. Through a comparison of experiment results, a suitable frequency range for time–frequency transform can improve fault diagnosis accuracy obviously. In IG-CWT, the CWT is used for time–frequency transform, but some other time–frequency transform may also work, for e.g., S-transform.

The limitation of our method is that the CNN model needs to be trained two times, once for data preprocessing and once for fault diagnosis, which is time consuming. As for future work, we will focus on how to incorporate data preprocessing into training processing of the CNN model.

## Figures and Tables

**Table 1:** Results of different λ on PU.

λ	AlexNet		ResNet18		VGG16	
−	$F_{range}$ (kHz)	Acc (%)	$F_{range}$ (kHz)	Acc (%)	$F_{range}$ (kHz)	Acc (%)
1	0.4–9.9	97.67 ± 0.17	0.6–10.1	98.41 ± 0.35	0.2–10.1	97.20 ± 0.20
0.7	0.4–10.2	97.89 ± 0.25	0.6–10.3	98.42 ± 0.19	0.2–10.3	97.51 ± 0.12
0.6	0.4–10.5	98.13 ± 0.21	0.4–11.5	98.90 ± 0.19	0.2–11.2	97.82 ± 0.31
0.5	0.4–11.1	98.29 ± 0.25	0.4–12.9	99.18 ± 0.37	0.3–12.0	97.81 ± 0.23
0.4	0.4–11.2	98.28 ± 0.10	0.4–13.2	99.17 ± 0.35	0.3–12.9	97.98 ± 0.14
0.35	0.4–12.2	98.46 ± 0.27	0.4–15.3	99.38 ± 0.31	0.2–16.6	**98.67 ± 0.26**
0.3	0.4–12.3	98.51 ± 0.28	0.4–17.0	**99.54 ± 0.17**	0.2–19.0	98.53 ± 0.28
0.25	0.2–16.8	**98.61 ± 0.32**	0.2–17.4	99.52 ± 0.21	0.2–19.1	98.58 ± 0.12
0.2	0.2–25.1	98.42 ± 0.15	0.2–22.8	99.23 ± 0.21	0.2–23.6	98.25 ± 0.14

**Table 2:** Results of different *λ* on MFPT.

λ	AlexNet		ResNet18		VGG16	
−	$F_{range}$ (kHz)	Acc (%)	$F_{range}$ (kHz)	Acc (%)	$F_{range}$ (kHz)	Acc (%)
1	1.0–18.7	89.04 ± 0.33	0.7–17.0	92.23 ± 0.45	1.1–17.7	92.23 ± 0.23
0.7	0.9–18.8	89.21 ± 0.25	0.5–17.4	92.24 ± 0.17	0.8–18,7	92.31 ± 0.34
0.6	0.9–19.0	89.12 ± 0.21	0.5–17.7	92.22 ± 0.23	0.7–18.7	92.69 ± 0.21
0.5	0.7–19.3	90.41 ± 0.31	0.5–17.9	92.32 ± 0.27	0.7–18.7	92.69 ± 0.21
0.4	0.5–19.4	91.32 ± 0.10	0.5–18.1	92.99 ± 0.35	0.5–18.9	93.45 ± 0.14
0.35	0.5–19.4	91.32 ± 0.10	0.5–18.3	93.38 ± 0.23	0.5–18.9	93.45 ± 0.14
0.3	0.4–20.0	**91.87 ± 0.32**	0.4–20.5	**94.00 ± 0.12**	0.5–19.1	**94.52 ± 0.24**
0.25	0.4–20.0	**91.87 ± 0.32**	0.4–20.5	**94.00 ± 0.12**	0.5–19.4	93.33 ± 0.22
0.2	0.2–22.1	91.32 ± 0.37	0.3–21.5	91.67 ± 0.43	0.5–23.1	91.21 ± 0.25

**Table 3:** Results of different *λ* on JNU.

λ	AlexNet		ResNet18		VGG16	
−	$F_{range}$ (kHz)	Acc (%)	$F_{range}$ (kHz)	Acc (%)	$F_{range}$ (kHz)	Acc (%)
1	0.4–1.1	97.25 ± 0.17	0.3–3.3	99.14 ± 0.25	0.3–2.9	97.51 ± 0.22
0.7	0.3–1.6	97.50 ± 0.08	0.3–5.0	99.25 ± 0.12	0.3–3.2	97.85 ± 0.32
0.6	0.3–4.2	97.43 ± 0.21	0.3–5.8	99.31 ± 0.18	0.3–5.1	98.36 ± 0.26
0.5	0.3–7.8	98.19 ± 0.15	0.3–7.7	99.18 ± 0.37	0.3–8.2	98.41 ± 0.13
0.4	0.3–8.0	98.28 ± 0.26	0.3–8.1	99.22 ± 0.25	0.3–8.5	98.45 ± 0.24
0.35	0.3–8.2	98.46 ± 0.19	0.3–9.0	**99.74 ± 0.12**	0.3–8.7	98.56 ± 0.18
0.3	0.3–8.8	**98.71 ± 0.23**	0.3–10.3	99.49 ± 0.27	0.3–9.3	**99.05 ± 0.15**
0.25	0.3–9.9	98.42 ± 0.30	0.2–11.4	99.48 ± 0.22	0.2–10.3	98.78 ± 0.27
0.2	0.2–21.1	98.21 ± 0.15	0.2–22.8	99.23 ± 0.31	0.2–19.9	98.25 ± 0.28

**Table 4:** Results of different λ on CWRU.

λ	AlexNet		ResNet18		VGG16	
−	$F_{range}$ (kHz)	Acc (%)	$F_{range}$ (kHz)	Acc (%)	$F_{range}$ (kHz)	Acc (%)
1	0.2–2.3	98.41 ± 0.12	0.3–2.4	98.48 ± 0.21	0.2–2.2	98.03 ± 0.34
0.7	0.2–3.0	98.67 ± 0.21	0.2–2.9	98.79 ± 0.16	0.2–2.8	98.82 ± 0.15
0.6	0.2–3.4	98.89 ± 0.14	0.2–3.4	99.15 ± 0.14	0.2–3.3	98.98 ± 0.21
0.5	0.1–3.8	99.11 ± 0.18	0.2–3.7	99.18 ± 0.12	0.2–3.5	98.95 ± 0.11
0.4	0.1–3.9	99.10 ± 0.19	0.1–4.0	99.22 ± 0.21	0.1–3.9	99.31 ± 0.15
0.35	0.1–4.2	99.29 ± 0.21	0.1–4.6	**99.87 ± 0.20**	0.1–4.2	99.38 ± 0.18
0.3	0.1–4.5	**99.61 ± 0.09**	0.1–4.7	99.77 ± 0.11	0.1–4.5	99.49 ± 0.17
0.25	0.1–4.8	99.42 ± 0.13	0.1–5.0	99.45 ± 0.24	0.1–4.6	**99.51 ± 0.18**
0.2	0.0–5.2	99.02 ± 0.24	0.0–5.4	99.14 ± 0.16	0.0–5.1	99.19 ± 0.28

## Data Availability

The data are publicly available.
